# Survival of Human Norovirus Surrogates in Water upon Exposure to Thermal and Non-Thermal Antiviral Treatments

**DOI:** 10.3390/v12040461

**Published:** 2020-04-19

**Authors:** Shu Zhu, Candace Barnes, Sutonuka Bhar, Papa Hoyeck, Annalise N. Galbraith, Divya Devabhaktuni, Stephanie M. Karst, Naim Montazeri, Melissa K. Jones

**Affiliations:** 1 Department of Molecular Genetics and Microbiology, University of Florida, Gainesville, FL 32611, USA; shuzhu@zju.edu.cn (S.Z.); divyadev92@gmail.com (D.D.); skarst@ufl.edu (S.M.K.); 2 Department of Food Science and Human Nutrition, IFAS, University of Florida, Gainesville, FL 32611, USA; candacebarnes@ufl.edu (C.B.); nmontazeri@ufl.edu (N.M.); 3 Department of Microbiology and Cell Science, IFAS, University of Florida, Gainesville, FL 32611, USA; sutonuka.bhar@ufl.edu (S.B.); phoyeck@ufl.edu (P.H.); annalisegalb@ufl.edu (A.N.G.)

**Keywords:** antiviral treatment, chitosan microparticles, disinfection, enteric virus, food safety, surface water, norovirus, public health, surrogates

## Abstract

Human noroviruses are the leading cause of foodborne gastroenteritis worldwide and disease outbreaks have been linked to contaminated surface waters as well as to produce consumption. Noroviruses are extremely stable in water and their presence is being detected with increasing frequency, yet there are no viable methods for reducing norovirus contamination in environmental water. Despite this, there is little knowledge regarding the physical and chemical factors that influence the environmental persistence of this pathogen. This study evaluated the impact of common chemical and physical properties of surface water on the stability of murine norovirus and examined the effect of food-safe chitosan microparticles on infectivity of two human norovirus surrogates. While chemical additives had a minor impact on virus survival, chitosan microparticles significantly reduced infectious titers of both murine norovirus and MS2 bacteriophage.

## 1. Introduction

Human noroviruses are a leading cause of diarrheal disease from unsafe food worldwide [1] and are responsible for >58% of the approximately nine million total cases of foodborne disease each year in the U.S [2]. Survey studies of the U.S and Europe have estimated that human noroviruses account for 59% of produce-related outbreaks [3] and these viral genomes are repeatedly detected on market-ready produce [4,5,6,7]. In addition, untreated recreational waters have been linked to norovirus disease outbreaks [8,9,10]. The presence of human pathogens in environmental waters that are used for drinking, recreation, and agricultural use is of increasing concern in the U.S. due to their ability to facilitate disease outbreaks [3,11,12,13]. Contamination of these waters by enteric pathogens can occur in several ways including discharge of treated and untreated wastewater, illegal dumping of human excrements, and unintentional discharges due to urban, rural and agricultural run-off [14,15]. As a result, preventing entry of enteric pathogens into these water sources is not always possible, and finding viable and cost-effective methods for eliminating contamination is needed. 

Human norovirus contamination of surface waters is of particular concern due to the low infectious dose of the virus and its ability to persist for long periods of time outside of the host [16,17,18]. Specifically, these viruses are stable in groundwater, river water, mineral water, and tap water for weeks to months [19,20,21]. Several factors such as hardness, solar radiation and phosphate levels, have also been suggested to influence virus survival in the water column [22,23,24,25]. The increased use of fertilizers has also led to a rise in chemical concentrations in natural water systems [26,27,28], but the impacts of these chemicals, particularly ammonium and phosphates, on pathogen survival have not been widely studied [29,30,31,32]. 

Due to the long-term stability of human noroviruses in the environment, an effective, economical method for eliminating the virus from surface water and produce wash water is needed. As a result, several thermal and non-thermal strategies have been investigated to inactivate human norovirus, but the results have not always been conclusive [33,34]. Some impeding factors include: (1) the lack of a robust human norovirus cell culture system to directly assess the efficacy of the treatments, (2) the variability in the response of human norovirus surrogate viruses to the disinfectant; (3) limited comprehensive studies on the response of surrogates to multiple antiviral treatments; and (4) variability in experimental conditions such as virus preparation, temperature, virus suspension matrix, and the variability in chemical formulation of the disinfectants [35]. 

To address the issue of norovirus environmental contamination, many methods are currently being investigated for their ability to inactivate the pathogen in water and on produce. One natural compound with demonstrated anti-microbial activity is chitosan. Chitosan is a carbohydrate polymer, a derivative of chitin, which is a naturally abundant carbohydrate obtained from shrimp shells and fungi. Chitosan is recognized for its broad antibacterial activity with industrial and medical applications [36,37,38,39], but its activity in acidic conditions limits its application in the natural environment. Studies investigating the impact of chitosan on stability of human norovirus surrogates have generated mixed results, and the effectiveness of the compound may be linked to particle size [40,41]. Recent advancements in generating chitosan particles at micro and nanoscales has enabled the successful in vivo and in vitro elimination of pathogenic bacteria in cattle [42] and in crop irrigation ponds [43]. Due to these enhanced properties, it was hypothesized that chitosan microparticles (CM) would be more effective against noroviruses than previously studied formulations of chitosan. 

This study investigated the impact of physical and chemical characteristics of surface water including ammonium, phosphate, and ultra-violet light, on the stability of murine norovirus in water at various temperatures. The antiviral activity of chitosan microparticles against murine norovirus and bacteriophage MS2, two broadly utilized human norovirus surrogates, was also examined. 

## 2. Materials and Methods

### 2.1. Eukaryotic Cell Lines, Bacterial Cells and Media

RAW 264.7 cells (ATCC 6003) and HEK-293T cells (ATCC 11268) were used to generate virus stocks. Both cell lines were cultivated in DMEM (Corning) supplemented with 10% FBS (Sigma) and 1× penicillin/streptomycin (Corning) and grown at 37 °C at 5% CO_2_. *Escherichia coli* C3000 (ATCC 15597) was cultured in tryptic soy broth medium at 37 °C. The *Salmonella* strain used in this study was an avirulent surrogate strain of *S. enterica* Typhimurium ATCC 14,028 with no pathogenicity islands and the *Salmonella* virulence plasmid [44]. *Salmonella* stocks were grown overnight in lactose broth at 37 °C.

### 2.2. Virus Stocks Preparation

Recombinant virus stocks of murine norovirus-1 (MNV-1) were generated as previously described [45]. Briefly, HEK-293T cells were transfected with 5 µg pSPMNV-1 using Lipofectamine 2000 (Life Technologies) and harvested at 24 h post-transfection. The cells were lysed by freeze-thaw at −80 °C and supernatants clarified by centrifugation. The recombinant virus was used to infect RAW 264.7 cells at an MOI of 0.05. Cell lysates were centrifuged through a sucrose cushion and the viral pellet resuspended, aliquoted and stored at −80 °C for future use. Full-length genomic sequences were determined for all recombinant virus stocks and confirmed to contain no deviations from the original clones. All MNV stocks were titrated by a standard TCID_50_ assay [46]. 

Bacteriophage MS2 (ATCC 15597-B1) were propagated by infecting log-phase growing cultures of *E. coli* C3000 at an MOI of 0.1 in tryptic soy broth medium. After 4–6 h of incubation at 37 °C, the cell debris was removed with centrifugation for 10 min at 10,000× *g*, then the supernatant was stored at −80 °C. The suspension was quantified by plaque assay and RT-qPCR and served as the inoculum stock [47,48].

### 2.3. Physical and Chemical Treatments

Sterile demineralized water was chemically adjusted to achieve variations in concentrations for phosphate and ammonium. Concentrations were confirmed using Hach Kits (Table 1). Each solution was inoculated with either high (10^7^ TCID_50_/mL) or moderate (10^4^ TCID_50_/mL) titers of MNV-1. Due to the known impact of temperature on virus stability [49,50], each chemical treatment was also evaluated at various temperatures (4 °C, 25 °C, 37 °C, and 60 °C). Virus suspensions were incubated at the stated temperatures and sampled at 0, 3, 7, 10, and 42 days post inoculation (dpi). To assess the impact of ultraviolet light on murine norovirus survival, high and moderate concentrations of virus were exposed varying doses of UV radiation (Table 1). Using a VWR UV-crosslinker, viral solutions were dosed for 0, 25 s, 4.2 min, and 10.4 min to yield total radiation exposures of 0, 10,000, 100,000 and 250,000 µJ/cm^2^ for the given samples. As a baseline control, untreated sterile demineralized water was inoculated in parallel for each condition. Virus was quantified using TCID_50_ assay. All experiments were performed in triplicate with two technical replicates in each experiment.

### 2.4. Treatment with Chitosan Microparticles

Chitosan powder (ChitoClear, TM4815) was procured from Primex, Iceland. According to the product information sheet, this formulation contains 91.4% dry weight, 0.4% ash, 90% degree of acetylation, 99.9% solubility, with a fine particle size (100% pass through 100 mesh). Chitosan microparticles (CM) were prepared according to Ma et al. [51] with minor modifications. Briefly, 10 g chitosan powders were dissolved in demineralized water containing 0.04% acetic acid and 1% Tween 80. Then, a 31 mL of 10% sodium sulfate was added dropwise while sonicating the chitosan solution, followed by centrifugation at 10,000× *g*, 10 min, 4 °C to remove the supernatant. This step was repeated two more times with demineralized water before suspending the chitosan microparticles in demineralized water. This CM suspension (6–10% dry weight) was stored at 4 °C in the dark and utilized as a stock. The minimum inhibitory concentration (MIC) was used to assess the reactivity and stability of each CM preparation. The MIC was performed by testing growth of the serially diluted CM (0.1% to 0.003125%, *w/v*) against the overnight culture of *Salmonella* and the MS2 host *E. coli* at 5 log CFU/mL following the incubation at 37 °C. The MIC was calculated based as the lowest concentration of CM that inhibited visible bacterial growth after overnight incubation.

To assess the antiviral activity of CM, different concentrations of CM in PBS (pH 7.4), were inoculated with aliquots of either MNV-1 (10^7^ TCID_50_/mL) or MS2 (10^10^ PFU/mL) and mixed gently at room temperature for a desired contact time. At the desired time points, a sample was taken from the suspension and virus survival was quantified using RT-qPCR and plaque assay. Positive control (inoculated PBS) and negative control (PBS only) were incorporated in all the experiments. Binding assay was performed separately by centrifuging the CM-virus reaction mixture at 10,000× *g* for 5 min and testing the titer of viral genome in the supernatant and pellet with RT-qPCR.

### 2.5. RT-qPCR

MNV-1 RNA was extracted using RNA-miniprep (Zymo), and MS2 RNA was isolated utilizing the Monarch Total RNA miniprep kit (New England Biolabs), all according to the manufacturer instructions. Viral RNAs were stored at −80 °C. MNV RNA was converted to cDNA using the IM-Prom II system (Promega) using 500 ng of RNA for each sample and following the manufacturer’s instructions. qPCR was performed using SYBR Green (Thermo Fisher) and primers for VP1 (sense 5′-CTTTGGAACAATGGATGCTG-3′ and antisense 5′-CGCCATCACTCATCCTCAT-3′) [52]. Each cDNA sample was amplified in triplicate. To determine viral genome copy, standard curves were generated using serial dilutions of pSPMNV-1 (Figure S1). 

Viral MS2 genome was used as RNA standard for the quantification of virus survival (Figure S2). A Luna Universal Probe One-Step RT-qPCR Kit (NEB) master mix with MS2F (TGGCACTACCCCTCTCCGTATTCACG) and MS2R (GTACGGGCGACCCCACGATGAC) primers and MS2P (Cy5-CACATCGATAGATCAAGGTGCCTACAAGC-BHQ2) probe was used for the amplification of MS2 RNA using CFX96 Touch real-time PCR detection system (Bio-Rad, Hercules, CA, USA) [48]. Viral RNA was reverse-transcribed for 10 min at 55 °C. After activating the Hot Start *Taq* DNA polymerase for 1 min at 95 °C, thermal cycling was carried out for 15 s at 95 °C, 1 min at 56 °C, and 1 min at 60 °C for a total of 45 cycles. Viral RNA was semi-quantified by comparing the cycle threshold values (Ct) of serially diluted viral RNA from standard curve to that of experimental samples. To determine viral genome copy number, standard curves were generated using decimal dilutions of MS2 RNA (Figure S2).

### 2.6. TCID_50_ Assay

For chemical, thermal, and UV treatments, MNV-1 was enumerated using a TCID_50_ assay [46]. Briefly, RAW 264.7 cells were seeded into 96-well plates and incubated overnight at 37 °C and 5% CO_2_. Samples were serially diluted in culture media and each dilution was plated into eight wells. Plates were incubated at 37 °C and 5% CO_2_ for seven days. Following incubation, wells were examined under an inverted microscope and scored for presence or absence of cytopathic effects, and the Reed-Muench method [53] was used to determine TCID_50_/mL.

### 2.7. Viral Plaque Assays

To quantify infectious virus from CM experiments, plaque assays were performed. For MNV-1, RAW 264.7 cells were seeded into six-well plates and incubated overnight at 37 °C and 5% CO_2_. Samples were serially diluted in complete DMEM, plated in duplicate wells, and plates incubated for 1 h at room temperature while rocking. The inoculum was then aspirated, and the cells overlaid with 1.5% SeaPlaque (Lonza) agarose in MEM supplemented with 10% FBS. Plates were incubated at 37 °C and 5% CO_2_ for 3 d. Plaques were visualized by overlaying with 2 mL of 1.5% SeaKem (Lonza) agarose containing 1% neutral red per well for 5 h [54]. 

Infectious titer of bacteriophage MS2 was quantified with a double-layer agar method using the *E. coli* strain C3000 (ATCC 15597) as host [47]. Following the treatment with chitosan microparticles, 700 µL of the serially diluted MS2 were mixed with 300 µL of log-phase growing *E. coli* and 8 mL top agar (tryptic soy broth with 0.6% agar), then overlaid on a bottom agar (tryptic soy broth with 1.2% agar). Plaque forming units (PFU) were counted following 24 h incubation at 37 °C.

### 2.8. Data Analysis

All data were presented as the mean of three replicates (mean ± SD). Statistical analyses were carried out using the analysis tool in GraphPad Prism. Specifically, three-way ANOVA and multiple *t*-test analyses were used to determine significant differences among treatments. Significant differences among the treatments were tested using a One-Way ANOVA followed by a Tukey’s HSD test for pair-wise comparisons among the categories. For all experiments, a *p*-value of <0.05 was considered statistically significant. 

## 3. Results

### 3.1. Effect of Physical and Chemical Parameters on In Vitro Survival of MNV

**Figure 1:**
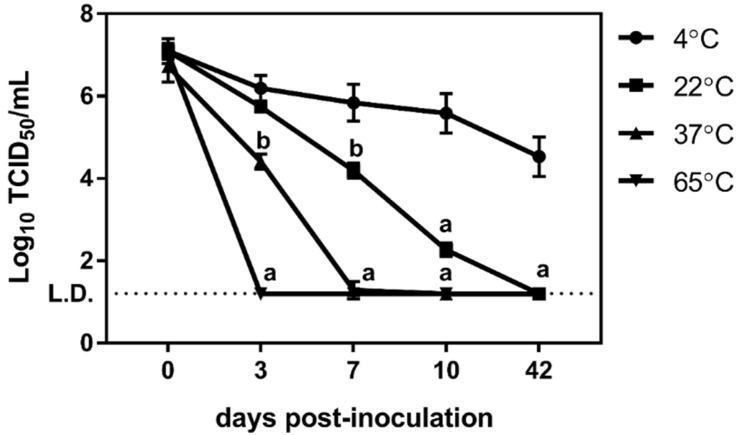
Stability of murine norovirus during extended incubation in DI water at various temperatures. Incubation of murine norovirus at various temperatures in DI water over 42 days resulted in significant (*p* ≤ 0.03) decreases in infectious virus at all temperatures. Viral titers decreased more rapidly at higher temperatures. a: *p* ≤ 0.0002, b: *p* ≤ 0.04, c: *p* ≤ 0.03.

The impact of common physical and chemical characteristics of surface waters on the long-term stability of murine norovirus was examined at 4 °C, 22 °C, 37 °C and 65 °C over 42 days. Prior to assessing stability as a result of the water conditions, the baseline stability of the virus in untreated water at these temperatures was determined. As previously demonstrated by others, the stability of murine norovirus is inversely correlated with temperature (Figure 1, [55,56]). After three days of incubation, significant (*p* ≤ 0.05) decreases in virus concentration occur at the two highest temperatures and by day seven virus concentrations at the three highest temperatures are significantly less than concentrations at 4 °C (Figure 1). While a significant (*p* ≤ 0.05) 2.5-log reduction in virus over time occurred at 4 °C, by day 42, high titers of infectious virus were still present.

**Figure 2:**
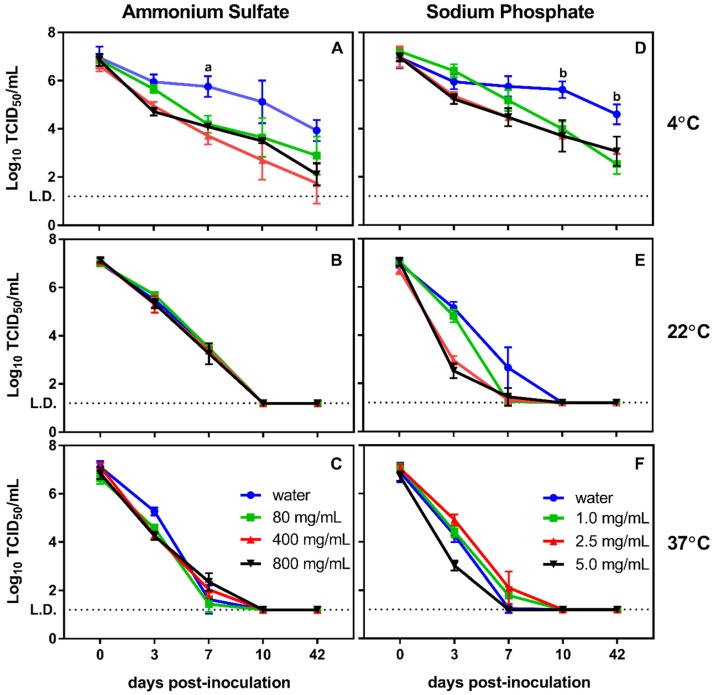
Stability of high murine norovirus titers during extended incubation upon exposure to ammonium sulfate and sodium phosphate. (**A**–**C**) Incubation of murine norovirus at various temperatures in the presence of ammonium sulfate over 42 days resulted in a temperature-dependent effect of the chemicals on murine norovirus stability. Increasing the temperature resulted in decreasing effectiveness of the chemical compounds against murine norovirus. However, at lower temperatures, even low concentrations of ammonium sulfate resulted in significant (a: *p* ≤ 0.003) decreases in culturable virus after one week of incubation and sodium phosphate reduced viral concentrations to undetectable levels. (**D**–**F**) similar results were also observed for sodium phosphate where increasing temperature resulted in decreasing effectiveness of the compound. At lower temperatures, all concentrations of sodium phosphate produced significantly (b: *p* ≤ 0.002) reduced viral titers.

We examined the impact of ammonium chloride and sodium phosphate on murine norovirus stability over time, using previously tested temperatures and two virus inoculum concentrations. When inoculated with high viral titers (10^7^ TCID_50_/mL), ammonium sulfate did not significantly impact virus stability at room temperature or 37 °C compared to untreated controls (Figure 2B,C). However, ammonium chloride treatment at 4 °C resulted in reduced virus survival beginning at seven days post inoculation, with the two highest concentrations of ammonium chloride (400 and 800 mg/mL) reducing viral titers to near the limit of detection by 42 days (Figure 2A). In contrast to ammonium chloride, incubation in sodium phosphate resulted in significant reductions in viral titer at 22 °C after seven days of incubation in 2.5 and 5 mg/mL. Moreover, 5.0 mg/mL of sodium phosphate reduced viral titers more rapidly than the untreated control at 37 °C (Figure 2E,F). At 4 °C, the chemical treatment also resulted in a decrease in viral titers over time, but the virus remained detectable through the end of the experiment (Figure 2D).

**Figure 3:**
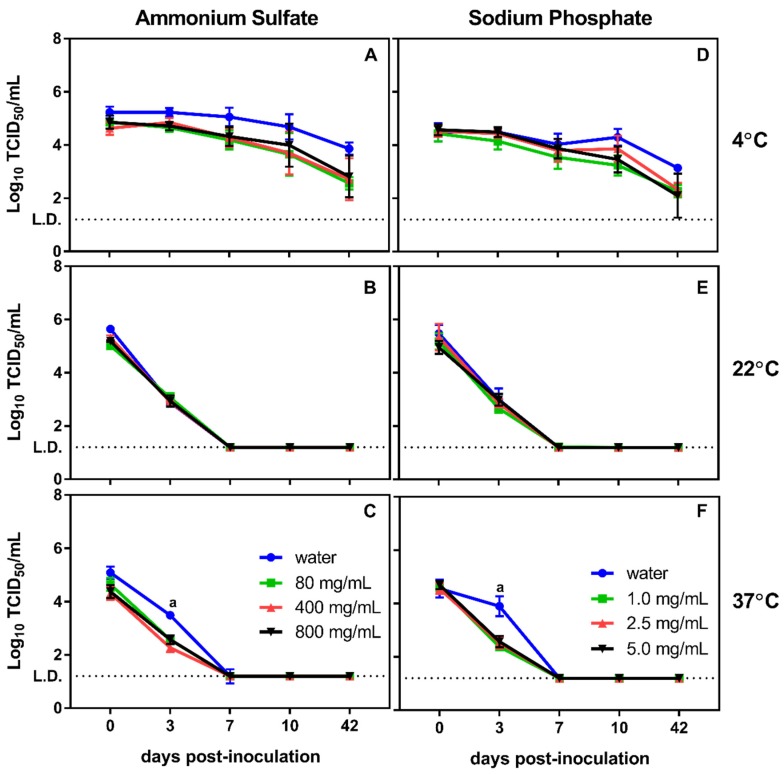
Stability of low murine norovirus titers during extended incubation upon exposure to ammonium sulfate and sodium phosphate. (**A**–**C**) Incubation of murine norovirus in the presence of ammonium sulfate at various temperatures over 42 days reduced viral titers to undetectable levels by day seven at 22 °C and 37 °C. At 37 °C, treatment with any concentration of ammonium sulfate resulted in a significant (a: *p* ≤ 0.02) reduction in viral titers by day three compared to the untreated control. (**D**–**F**) Similar results were also observed for sodium phosphate and with this compound significant (a: *p* ≤ 0.02) decreases in viral titers were only observed after 3 days of incubation at 37 °C Decreases in viral titers were not enhanced by the presence of either chemical reagent regardless of temperature.

Given that concentrations of human norovirus detected in environmental water are dramatically lower than those detected in fecal samples, the impact of ammonium sulfate and sodium phosphate were tested against lower input inoculums of murine norovirus (10^4^ TCID_50_/mL). At these lower titers, virus survival in the presence of these compounds was similar to that of the untreated controls at all time points when incubated at 4 °C and 22 °C (Figure 3A,B and Figure 3D,E). At 37 °C, both compounds displayed significantly lower viral titers after three days of incubation compared to the untreated control (Figure 3) indicating a potential synergistic increase in viral degradation. Unlike high murine norovirus titer experiments, virus titers dropped to undetectable levels by seven days at both 22 °C and 37 °C, regardless of treatment conditions. However, at 4 °C, virus was still detectable after 42 days of incubation (Figure 3).

**Figure 4:**
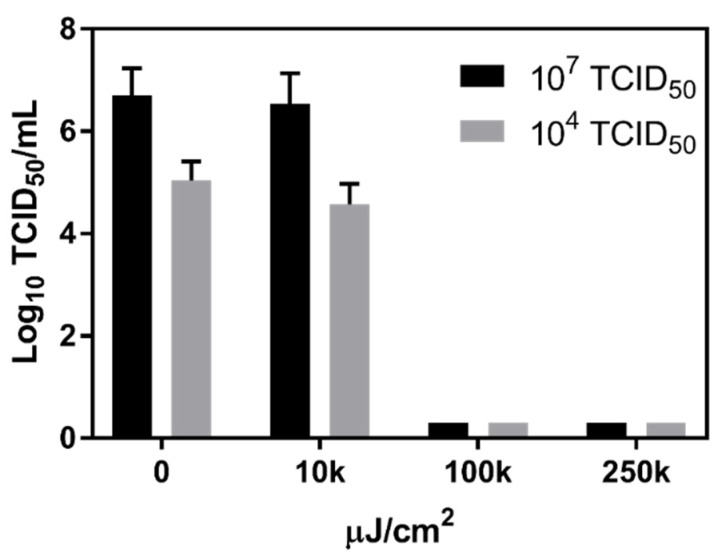
Stability of murine norovirus upon exposure to UV light. High and moderate titers of murine norovirus were exposed to UV light. For both titers, infectious virus was completely eliminated using 100,000 µJ/cm^2^ of UV light, while 10,000 µJ/cm^2^ did not result in a significant decrease in culturable virus for either at a high or moderate titer.

In addition to evaluating the impact of chemical constituents of surface water on murine norovirus survival, the impact of ultra-violet (UV) light on viral stability was also assessed. These experiments exposed both high (10^7^ TCID_50_) and moderate (10^4^ TCID_50_) viral titers to increasing intensities of UV light. Results showed that, regardless of input virus concentration, 10,000 µJ/cm^2^ did not significantly reduce the amount of infectious virus. However, UV light intensities ≥100,000 µJ/cm^2^ were sufficient to reduce viral titers below detection for both input concentrations (Figure 4). 

### 3.2. Determining the Impact Chitosan Microparticles on Human Norovirus Surrogates

**Figure 5:**
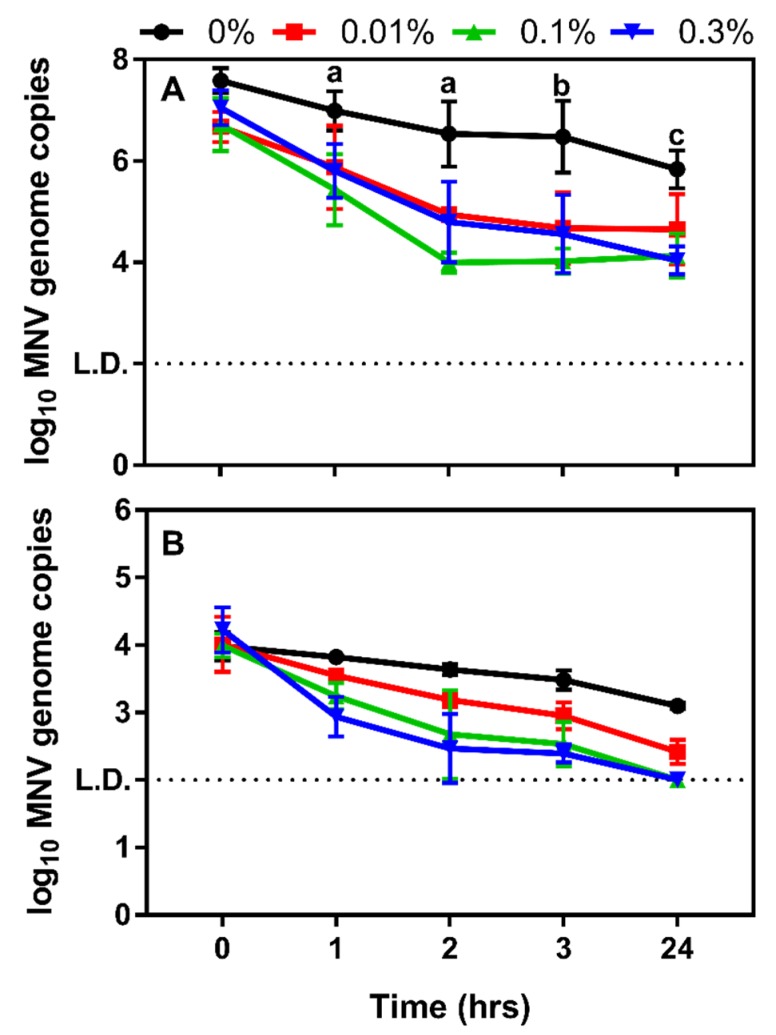
Murine norovirus survival after CM treatment. (**A**) High doses of murine norovirus (10^7^ TCID50/mL) were treated with varying concentrations of CM resulting in significant decreases in virus survival after 1 h of incubation with higher CM doses (0.1% and 0.3%, *p* ≤ 0.04). Increased incubation time resulted in significant decreases in murine norovirus genome at all tested CM concentrations (*p* ≤ 0.01). a: *p* ≤ 0.04, b: *p* ≤ 0.03, c: *p* ≤ 0.003; (**B**) At moderate doses of input murine norovirus (10^4^ TCID_50_/mL), significant decreases in viral genomes were not observed until 3 h post-inoculation, but the two highest concentrations of CM reduced viral titers to undetectable levels.

To determine the impact of CM on norovirus survival, high and moderate concentrations of murine norovirus were exposed to concentrations of CM ranging from 0.01 to 0.3%. A previous study evaluating the impact of 0.3% chitosan on murine norovirus survival revealed this compound was unable to significantly reduce viral titers [40]. However, treating murine norovirus with either 0.1 or 0.3% CM caused a significant decrease (*p* < 0.05) in viral genome copies after 1 h of incubation when a high inoculum of murine norovirus was used (Figure 5A). Extended incubation at these CM concentrations resulted in a 3-log reduction in viral genome titer while the titers of untreated samples decreased by less than a 2-log in the same time (24 h). Treatment of moderate viral inoculum did not result in significant reductions in viral genomes until 3 h post-inoculation (Figure 5B). However, increasing CM concentrations to 0.1% and 0.3% and increasing contact time to 24 h resulted in a reduction of murine norovirus genome to undetectable levels, which was not observed with higher concentrations of input virus (Figure 5A,B).

Based on the effectiveness of CM against murine norovirus, we performed dosing studies to determine the minimum inhibitory concentration (MIC) needed to reduce titers of the infectious virus. Given that plaque assays were used in previous chitosan studies to evaluate the effectiveness of the compound on survival of infectious virus, plaque assays were also used in assessing the impact of CM on both MNV and MS2. Prior to evaluating the impact of CM on infectious virus for either murine norovirus or MS2 bacteriophage, the impact of the compound on RAW 264.7 cells (used for murine norovirus plaque assay) and *E. coli* C3000 (ATCC 15597; used for MS2 bacteriophage plaque assays [42]) was assessed. Previous studies did not report any negative impact for a 3-h incubation of 0.07% chitosan oligosaccharide (5 kDa) to CRFK and RAW 264.7 cells [57]. To reduce the concentration of CM below the limit to negatively impact the plaque assay performance, CM-treated virus suspensions were diluted prior to applying to RAW 264.7 and *E. coli* cells. When compared with negative and positive controls, the plaque assays did not indicate any inhibitory effect of CM against the host cells (data not shown). Due to a previous report on the antimicrobial activity of CM against *E. coli*, an MIC for CM was first tested against the host *E. coli*. We also determined the MIC of CM for *Salmonella* serovar Montevideo to compare the antibacterial efficacy of our product with the same formulation reported elsewhere [43]. In our study, the respective MICs for the *E. coli* and *Salmonella* were 0.006% and 0.008% (*w/v*). Even at higher CM concentrations, we did not observe any interference of CM in the performance of plaque assay, as 0.4% CM was added directly to the top agar with no noticeable impact on *E. coli* growth. However, to eliminate the possibility of any negative impact of residual CM against *E. coli*, plaque assays evaluating the impact of CM on MS2 were performed after an initial decimal dilution of samples in PBS. This approach resulted in a CM concentration lower than the MIC against the *E. coli*.

**Figure 6:**
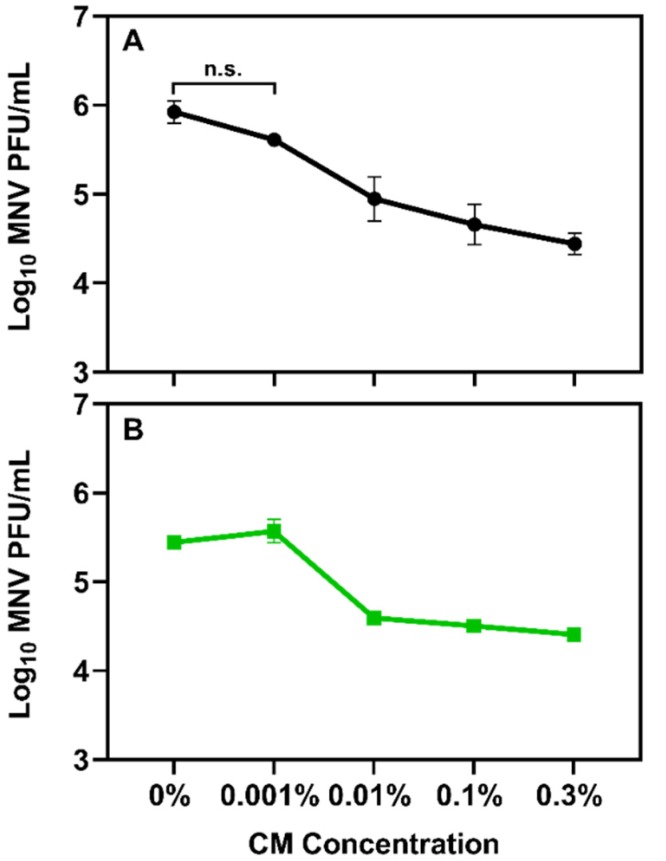
Minimum inhibitory concentration of CM against murine norovirus. (**A**) Dosing studies revealed that with high input titers of murine norovirus, at least 0.01% CM was needed to observe a significant reduction (*p* ≤ 0.01) in infectious murine norovirus titers compared to untreated controls after 1 h of treatment. (**B**) When moderate input titers of murine norovirus were exposed to CM, 0.01% resulted in a significant reduction in infectious virus. Increasing concentrations of CM did not result in a significant reduction in viral titers after 1 h of incubation.

Given that the experimental concentrations of CM were not deleterious to RAW 264.7 macrophages, the MIC for murine norovirus was determined. Dilutions of CM were mixed with 10^7^ TCID_50_/mL of virus and the mixtures were sampled immediately and again after 1 h of incubation. Results showed that a concentration of 0.001% did not result in significant decreases in infectious virus concentration at either time point (Figure 6A,B). Interestingly, at all the other tested concentrations, an immediate, dose-dependent decrease in infectious virus was observed where 1.1, 1.3, and 1.5 log PFU reductions in virus titers were observed for 0.01%, 0.1% and 0.3% (*w/v*) CM, respectively (Figure 6A). 

**Figure 7:**
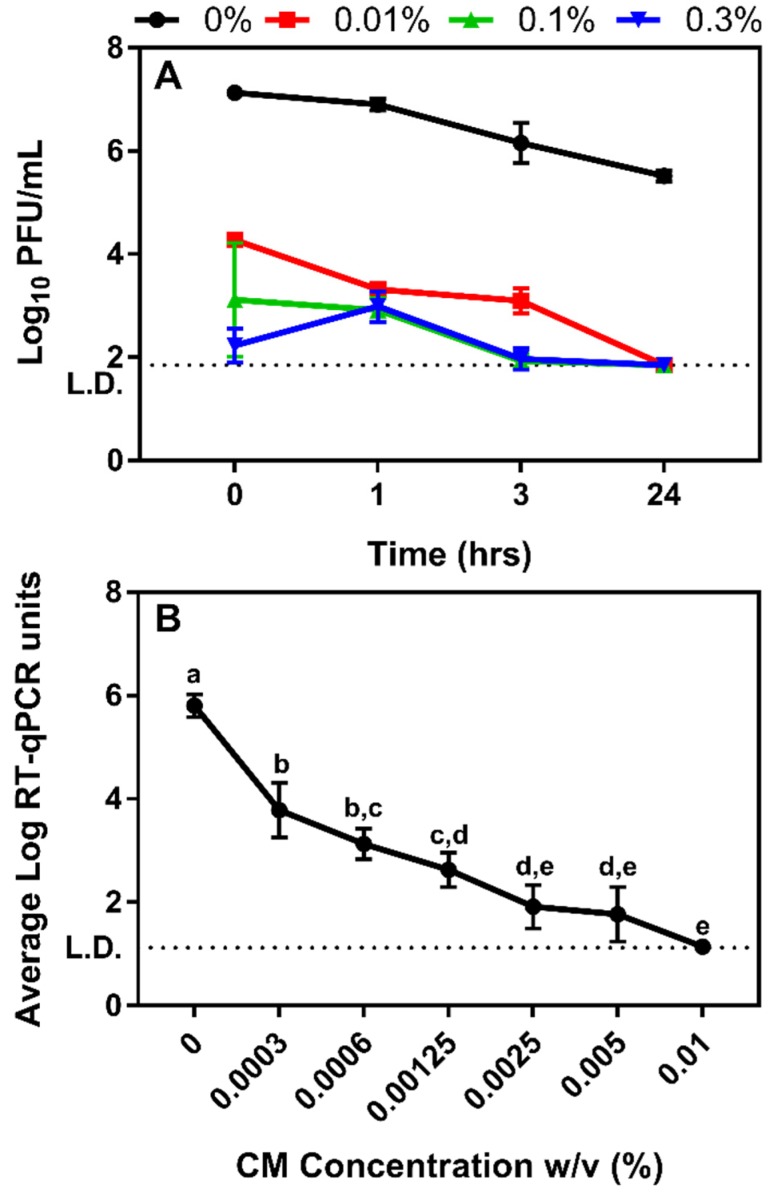
Impact of CM on MS2 survival as determined with plaque assay and RT-qPCR. (**A**) The impact of CM was immediate on MS2 infectivity and all tested concentrations resulted in immediate and significant decreases in titer. At 0.3% CM, viral titer was significantly reduced to 2.2 log PFU/mL (*p* < 0.0001). Values reported as mean ± SD, with a 1.85 log PFU/mL limit of detection. (**B**) The impact of CM was immediate on MS2 and all tested concentrations resulted in immediate and significant decreases in MS2. Viral genome decreased by approximately 4.0 log RT-qPCR units up to 0.0025% CM at 0-h contact time (a–e: *p* < 0.0001). Increasing CM concentration to 0.01% resulted to almost a complete loss of RT-qPCR signal (a–e: *p* < 0.0001). Values reported as mean ± SD. CM concentrations not connected by same letter are significantly different. Dashed line shows the 1.12 log RT-qPCR units limit of detection.

Similar experiments testing the antiviral activity of CM were also performed using MS2 bacteriophage, where the impact of CM up to 0.3% (*w/v*) was tested against the virus for up to 24 h of contact time. These results showed that immediate contact with 0.01% CM resulted in a 2.9 log PFU reduction (*p* = 0.0013) in the infectious titer of MS2 (Figure 7A; *p* < 0.05). One hour of contact time yielded further decreases in MS2 titer and reached at or below the 1.85 log PFU/mL limit of detection after 24 h for all the CM concentrations tested (*p* < 0.001, Figure 7A). At all tested concentrations of CM, a significant decrease in infectivity was observed (b: *p* = 0.0013; bc: *p* = 0.0001; c: *p* < 0.0001). At 0.3%, viral titer reached the limit of detection of 1.85 log PFU/mL. Therefore, higher concentrations than 0.3% CM were not tested against MS2. 

Because of the large decrease in infectious MS2 titer observed upon immediate contact with 0.3% CM (Figure 7A), further dosing studies were performed to determine the concentration at which CM was no longer immediately effective against the virus. For these expanded assays, MS2 titers of CM- treated samples were measured using RT-qPCR. Similar to what was observed for plaque assay, all dilutions of CM resulted in an immediate decrease in MS2 titer (Figure 7B). At 0-h contact time, viral MS2 RNA showed a steady decrease from 3.8 to 1.9 log RT-qPCR units as the concentration of CM increased from 0.0003% to 0.0025%, and remained relatively unchanged up to 0.005% CM. Upon treatment with 0.01% CM, full inactivation was observed resulting in a 4.7 log reduction (*p* < 0.05) in MS2 viral genome in the samples.

**Figure 8:**
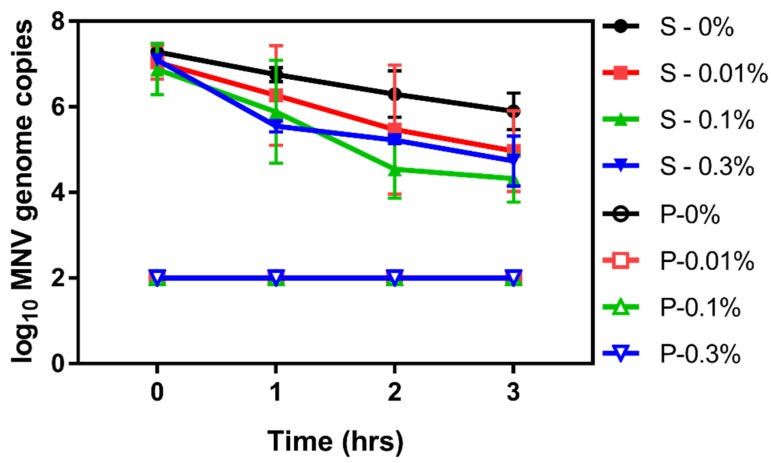
Binding of CM to murine norovirus. After incubation with murine norovirus, CM was pelleted and murine norovirus in pellets and supernatants were quantified using RT-qPCR. Results showed concentrations of murine norovirus bound to chitosan were below the limit of detection for all CM concentrations and at all time points and the majority of murine norovirus was found in the supernatant.

Given that the effectiveness of chitosan against bacterial agents has been linked to binding of the particles with the bacteria [39], we questioned if the same mechanism was responsible for reducing the viability of the norovirus surrogate viruses. Due to the immediate antiviral activity of CM against MS2 (Figure 7), further mechanistic studies with this virus were not possible, so experiments were conducted using only murine norovirus. To begin, binding assays were performed to determine if murine norovirus bound directly to CM by comparing viral titers in CM pellets compared to supernatant. Results showed that, unlike bacteria, the majority of murine norovirus was not bound to the CM particle but rather was found in the supernatant (Figure 8).

## 4. Discussion

Human noroviruses are recognized for their high environmental stability and low infectious dose [19,58]. Due to the enteric nature of the pathogen, this virus is released into sewage systems, and the conventional treatment of sewage does not sufficiently remove enteric viruses. One study detected up to 4 log titer of human norovirus GI and GII particles per mL in secondary-treated municipal wastewater in the United States [14]. Contaminated surface water has been linked to norovirus outbreaks after recreational use, and irrigation of vegetables and fruits [8,9,10,58]. Based on the need to eliminate noroviruses from surface or post-harvest wash waters, this study investigated the survival of human norovirus surrogates in water subjected to various chemicals, physical treatments and antibacterial chitosan microparticles.

As previously reported, and corroborated in this study, increasing temperatures and doses of ultra-violet light significantly decrease the survival of noroviruses [55,59]. In addition, ammonium sulfate and sodium phosphate were able to reduce viral titers. Both ammonium and phosphates have antimicrobial properties under specific conditions [24,60,61], and prolonged treatments with either of these compounds was able to reduce moderate titers of murine norovirus to undetectable levels. However, when incubated at 4 °C, both high and low concentrations of virus were still detectable even after 42 days of incubation. This indicates either a protective impact of low temperature or a synergistic effect of elevated temperatures and chemical treatment on viral stability. This impact of temperature on virus stability is an important consideration for stored produce items, while the impact of chemical treatments on moderate viral titers may be particularly applicable to environmental surface water in that concentrations of human norovirus detected in water or on produce are generally much lower than what is found in stool samples of infected individuals. The viral concentrations tested in these experiments mimic the environmental concentrations of norovirus and thus are promising for future downstream applications. 

For this reason, the impact of chitosan microparticles on the stability of human norovirus surrogate viruses was examined. Chitosan is an environmentally friendly carbohydrate biopolymer and is recognized for delivering a broad in vivo and in vitro antibacterial activity. For example, CM has been successfully applied for the mitigation of *Salmonella* in agricultural water [39,42,43]. The antiviral activity of CM was tested against murine norovirus and bacteriophage MS2 in PBS (pH 7.4). Chitosan has been recognized for having a low immunogenic activity and toxicity to mammalian cells [38,39] including the murine macrophages cells RAW 264.7 [62], which was confirmed in our studies. At high concentrations of CM, the antiviral activity of the microparticles was immediate on both surrogate viruses. CM at 0.3% resulted in a respective 0.9 and 4.7 log PFU reductions in murine norovirus and MS2 particles at 0-h contact time. Initial treatment with 0.01% CM (0-h contact time) did not appear to be effective against murine norovirus; however, it resulted in 2.9 log reduction in MS2 infectious particles. These results are consistent with previous studies showing a higher sensitivity of MS2 compared with murine norovirus to either a 222-kDa chitosan dissolved in acetic acid or a 53-kDa chitosan dissolved in water [40,41]. Considering the reduction in infectivity of MS2, the antiviral activity of CM against this virus does not seem to be improved over contact times. Similar effects were seen with murine norovirus, particularly with the more environmentally relevant viral titers, where extending contact time to 24 h did not dramatically improve viral reduction compared to 2–3 h of CM exposure. However, 24 h of contact time did reduce lower input titers of murine norovirus to non-detectable levels. Given the low concentrations of human norovirus typically detected in the environment and on foods, these results suggest CM is a promising candidate eliminating human norovirus contamination. 

The antiviral activity of chitosan is not completely elucidated. One study suggested that the loss of tail fibers, contraction of the tail sheath, and interaction of the basal plate proteins as possible mechanisms of inhibition of the T2 bacteriophage [63]. The interaction of positively charged chitosan with negatively charged functional groups on microbial cell membranes is believed to be a major contributor to the antibacterial activity of the compound [39]. Therefore, it was hypothesized that this interaction could contribute to the inactivation of chitosan-treated viruses due to the interaction of the chitosan with viral capsid proteins, which are exposed in non-enveloped viruses such as MS2 and murine norovirus. Interestingly, murine norovirus was not detected in the CM pellet and remained entirely in the supernatant, which suggests a different mechanism of action may exist for CM destruction of this virus. 

Even though the impact of CM on the integrity of viral RNA was not directly investigated in this study, it is anticipated that the reduction in viral genome was, at least partly, associated with the binding of the nucleic acids to the positively charged moieties of chitosan [39]. This hypothesis is based on the substantial impact of CM treatment on viral genomes. For example, CM at 0.0025%, resulted in almost full reduction of MS2 viral genome and reached a plateau thereafter until it reached at or below the 1.1 log RT-PCR unit limit of detection when applied at 0.01%. In the case of murine norovirus, however, no significant inactivation was observed until CM concentration reached to ≥0.1% and higher concentrations (≥0.01%) of CM were required to yield significant reductions of low titer murine norovirus. Even though MS2 appeared to be more sensitive than murine norovirus to CM treatment, the comparison should be cautioned due to the intrinsic variability of the surrogates in response to the antiviral treatments and difference in viral genome quantitation method utilized for the assessment of virus inactivation [35].

## 5. Conclusions

The results of this study conclude that enteric viruses could maintain their infectivity in water (<22 °C) for weeks. During this period, the virus particles are protected from strong UV light and low concentrations of ammonium and phosphate salts present in water. Furthermore, these studies demonstrated that low concentrations of CM are capable of lowering concentrations of human norovirus surrogate viruses, while high concentrations of CM can reduce viral titers to non-detectable levels. Increasing incubation time was found to be another effective means of reducing viral titers to non-detectable levels. While further studies are needed, the data presented herein, demonstrate that CM is a viable candidate for reducing or removing an environmentally stable viral pathogen from water sources.

## Figures and Tables

**Table 1:** Physicochemical parameters evaluated for impact on virus stability.

Category	Chemical Used	Concentration Range Tested
Temperature	N/A	4–65 °C
UV radiation	N/A	10,000–250,000 µJ/cm^2^
Phosphate	NaH_2_PO_4_	0–5 mg/L
Ammonium	NH_4_Cl	50–800 mg/L
Polysaccharide	Chitosan microparticles	0–0.3% (*w/v*)

## Supplementary Materials

The following are available online at https://www.mdpi.com/1999-4915/12/4/461/s1, Figure S1: Representative standard curve for determining murine norovirus concentration in samples quantified using RT-qPCR., Figure S2: Representative standard curve for RT-qPCR quantification of MS2 viral genome in samples.
