# Supplementary material for: Survival of Human Norovirus Surrogates in Water upon Exposure to Thermal and Non-Thermal Antiviral Treatments
Source: Viruses. 2020 Apr 19;12(4):461. doi: 10.3390/v12040461 (PMC7232373; doi:10.3390/v12040461)
Supplement: Supplementary file 1 [file viruses-12-00461-s001.pdf]

viruses

MDPI

Supplemental Results

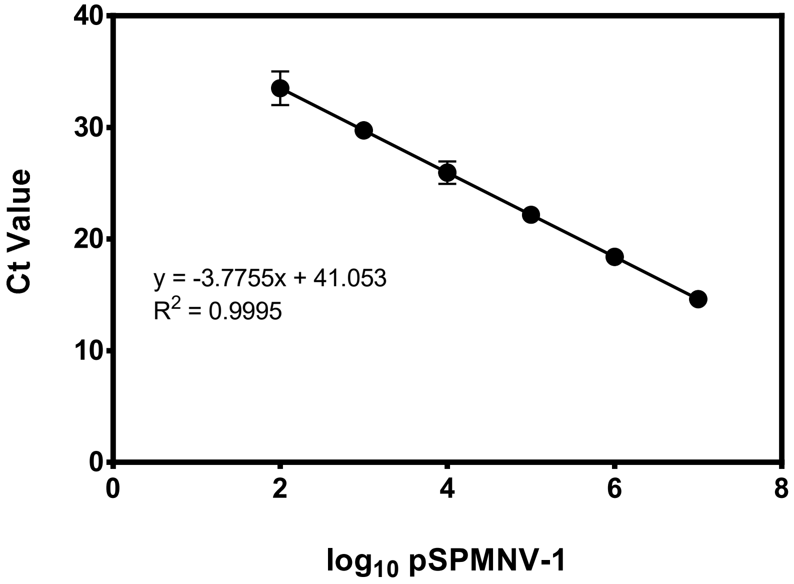

Figure S1. Representative standard curve for determining murine norovirus concentration in samples quantified using RT-qPCR. The plasmid pSPMNV-1 was used in qPCR analysis of MNV-1 containing samples to determine virus concentration. Serial dilutions starting from 10^7 and ending at 10^2 were used to generate the standard curve. Linear regression analysis of this curve resulted in an $R^2 = 0.9995$, which was typical for all RT-qPCR runs.

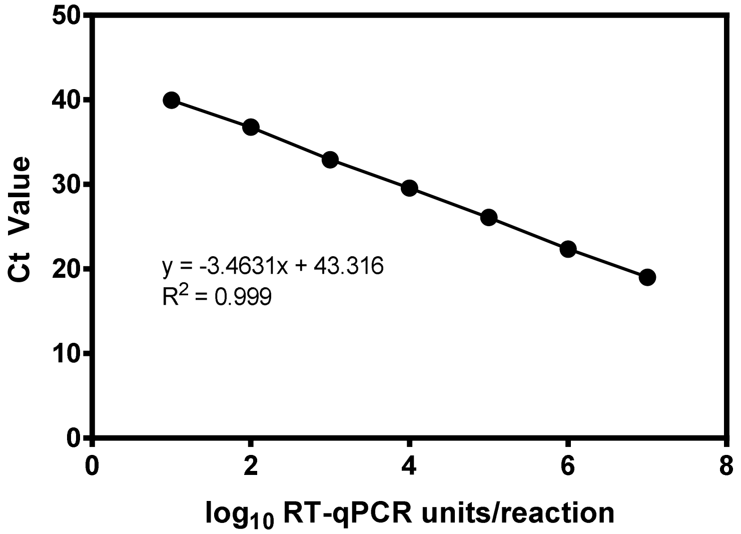

Figure S2. Representative standard curve for RT-qPCR quantification of MS2 viral genome in samples. The viral RNA was used for the determination of MS2 in the samples. The amplification signals showed a strong linearity ($R^2 = 0.999$) from 10^7 to 10^1 RT-qPCR Units.
